# Repulsive Guidance Molecule (RGM) Family Proteins Exhibit Differential Binding Kinetics for Bone Morphogenetic Proteins (BMPs)

**DOI:** 10.1371/journal.pone.0046307

**Published:** 2012-09-27

**Authors:** Qifang Wu, Chia Chi Sun, Herbert Y. Lin, Jodie L. Babitt

**Affiliations:** Program in Anemia Signaling Research, Nephrology Division, Program in Membrane Biology, and Center for Systems Biology, Massachusetts General Hospital, Harvard Medical School, Boston, Massachusetts, United States of America; Alexander Flemming Biomedical Sciences Research Center, Greece

## Abstract

Bone morphogenetic proteins (BMPs) are members of the transforming growth factor beta superfamily that exert their effects via type I and type II serine threonine kinase receptors and the SMAD intracellular signaling pathway to regulate diverse biologic processes. Recently, we discovered that the repulsive guidance molecule (RGM) family, including RGMA, RGMB, and RGMC/hemojuvelin (HJV), function as co-receptors that enhance cellular responses to BMP ligands. Here, we use surface plasmon resonance to quantitate the binding kinetics of RGM proteins for BMP ligands. We show that among the RGMs, HJV exhibits the highest affinity for BMP6, BMP5, and BMP7 with K_D_ 8.1, 17, and 20 nM respectively, versus 28, 33, and 166 nM for RGMB, and 55, 83, and 63 nM for RGMA. Conversely, RGMB exhibits preferential binding to BMP4 and BMP2 with K_D_ 2.6 and 5.5 nM respectively, versus 4.5 and 9.4 nM for HJV, and 14 and 22 nM for RGMA, while RGMA exhibits the lowest binding affinity for most BMPs tested. Among the BMP ligands, RGMs exhibit the highest relative affinity for BMP4 and the lowest relative affinity for BMP7, while none of the RGMs bind to BMP9. Thus, RGMs exhibit preferential binding for distinct subsets of BMP ligands. The preferential binding of HJV for BMP6 is consistent with the functional role of HJV and BMP6 in regulating systemic iron homeostasis. Our data may help explain the mechanism by which BMPs exert cell-context specific effects via a limited number of type I and type II receptors.

## Introduction

Bone morphogenetic proteins (BMPs) are a subfamily of the transforming growth factor beta (TGF-β) superfamily of signaling molecules that is comprised of over 40 members [1]. Although best characterized for their roles during development due to the frequent embryonic lethality or major organ malformation caused by functional loss of BMP/TGF-β signaling pathway components, there is an increasingly recognized role for BMP/TGF-β superfamily signaling during postnatal life [2].

BMP/TGF-β superfamily members exert their effects by binding to a complex of two type I and two type II serine threonine kinase receptors to stimulate phosphorylation of intracellular SMAD proteins, which complex with common-mediator SMAD4 and translocate to the nucleus to regulate gene transcription [1]–[2]. Thus far, 7 type I receptors (4 for the BMP subfamily) and 5 type II receptors (3 for the BMP subfamily) have been described. In general, BMPs signal via one subset of SMAD proteins (SMAD1, SMAD5, and SMAD8), while TGF-β ligands signal via another subset (SMAD2 and SMAD3) [1]–[2]. Other noncanonical signaling cascades can also be activated, but the molecular mechanisms are less well understood [1]–[2]. One important question in the field is how the BMP/TGF-β superfamily is able to exert a cell-context specific effect through a limited number of type I and type II receptors and intracellular SMAD proteins.

Recently, we discovered a novel family of proteins, the repulsive guidance molecule (RGM) family, that function as co-receptors for the BMP signaling pathway [3]–[5]. This family is comprised of 3 members, RGMA, RGMB, and RGMC (also known as hemojuvelin, hereafter referred to as HJV), that share 50–60% amino acid identity and similar structural features, including an N-terminal signal peptide, proteolytic cleavage site, partial von Willebrand factor type D domain, and glycophosphatidylinositol (GPI) anchor [6]–[11]. We have previously demonstrated that all RGMs bind to BMP ligands and BMP type I and type II receptors to enhance intracellular SMAD phosphorylation and BMP-SMAD target gene transcription in response to BMP ligands [3]–[5]. It has been hypothesized that expression of RGM proteins enables cells to selectively respond to low levels of BMP ligands [3]–[5]. At least some members of the RGM family have also been shown to be released from the cell in soluble form (lacking the GPI anchor) [8]–[9], [12]–[17], and soluble RGM proteins can act as inhibitors of the BMP signaling pathway by sequestering BMP ligands [3], [18]–[19].

RGM family members are differentially expressed in a wide range of tissues, and have been suggested to have diverse biologic roles ranging from repulsive axonal guidance (giving rise to the family name), neural tube closure, neuronal differentiation, cell survival, axonal regeneration after injury, immunity, inflammation, and iron homeostasis regulation [2]–[11], [20]–[33]. Some of these biologic actions depend on the BMP signaling function of RGM family members [5], [7], [18]–[21], [32]–[33], while others appear to be independent of the BMP signaling function of RGMs [23]–[24], [26]–[30].

HJV has the most well characterized biologic role among the RGM family that depends on its function as a BMP co-receptor. Mutations in the gene encoding HJV lead to the iron overload disorder juvenile hemochromatosis in both human patients and mice as a direct result of impaired BMP-SMAD signaling in the liver and consequent deficiency of the main iron regulatory hormone hepcidin [5], [7], [20]–[21]. The central role of the BMP-SMAD pathway in hepcidin regulation and systemic iron balance is further supported by the fact that mutations in the genes encoding the ligand BMP6 [18], [34], BMP type I receptors ALK2 and ALK3 [35], or common-mediator SMAD4 [36] all lead to hepcidin deficiency and iron overload similar to *HJV* mutations. Furthermore, pharmacologic modulators of the BMP-SMAD signaling pathway regulate hepcidin expression and systemic iron balance in normal mice [18]–[19], [37], and ameliorate iron overload due to hepcidin deficiency and anemia due to hepcidin excess in animal models [38]–[39].

Interestingly, indirect evidence from binding competition and biological inhibition assays using soluble RGM proteins lacking the GPI anchor fused to the Fc tail of human IgG (RGM.Fc) suggest that RGM proteins do not bind to all BMP ligands with equal affinity [3]–[5], [18]–[19], [40]. Biologic assays in cell culture systems also suggest that RGMs utilize some BMP ligands preferentially [4]–[5], [40]–[42]. The differential binding of RGMs to BMP ligands may provide insight into their biologic functions, and into the mechanisms by which BMPs generate a cell-context specific effect. Here, we used surface plasmon resonance (SPR) to quantitate the binding kinetics of RGM proteins to a wide array of BMP ligands including BMP2, BMP4, BMP5, BMP6, BMP7 and BMP9.

## Materials and Methods

### Surface Plasmon Resonance (SPR)

All SPR kinetics experiments were carried out on a Biacore T200 (GE Healthcare) except for BMP9, which was performed on an earlier model Biacore T100 (GE Healthcare). Recombinant human BMP2, BMP4, BMP5, BMP6, BMP7, BMP9, RGMA, RGMB, HJV (RGMC), and ALK1-Fc were obtained from R&D Systems in carrier-free forms. BMPs, RGMs and ALK1-Fc were reconstituted in sterile PBS as 1 µg/µl stock solutions, except for BMP6, which was supplied in Acetonitrile and TFA solution from R&D Systems at concentrations from 0.535 µg/µl to 0.788 µg/µl as indicated by the manufacturer.

BMP2, BMP4, BMP5, BMP6 and BMP9 were diluted in sodium acetate pH 4.5 (GE Healthcare) and immobilized by the amine coupling method on a CM5 sensor chip according to the manufacturer’s protocol (GE Healthcare). BMP7 was immobilized on a CM4 sensor chip using the same protocol. Flow cells 2 through 4 were immobilized with a ligand, and flow cell 1 was equally treated but without protein as a control. The immobilization levels of ligands ranged from 50 to 300 RU, determined empirically to optimize each kinetics interaction and reduce mass transport limitation. Low immobilization levels were used to minimize possible mass transport limitation, and at least 2 different immobilization levels were used for each interaction tested. In all cases, data obtained from different immobilization levels showed no significant difference. Analytes (RGMA, RGMB, HJV and ALK1-Fc) were diluted in running buffer HBS-EP+ (GE Healthcare) at concentrations ranging from 2 nM to 1200 nM, generally with a series of five 2-fold escalations to a maximum concentration about 10-fold higher than the K_D_, with occasional variations as needed to improve fitting. Analytes were injected through all channels at a flow rate of 30 µl/min, a flow rate chosen from a mass transport limitation test to minimize mass transport limitation, and above which further increases had no significant impact on binding curves. Middle concentrations were run in duplicate at the end of each multicycle run to confirm the stability of the surface during each run. The association and dissociation times were typically 240 seconds and 480 seconds, occasionally modified where indicated to optimize each interaction. The sensor surface was regenerated after each injection cycle to allow interaction between surface and fresh ligands for the next cycle. The regeneration condition was optimized by regeneration scouting as Glycine-HCl pH 2.2 (GE Healthcare) at a flow rate of 50 µl/min for 30 seconds followed by 30 seconds of stabilization.

The sensograms of flow cells 2, 3, and 4 were subtracted from the flow cell 1 control. The kinetic fitting was carried out with Biacore T200 evaluation software by global fitting using 1∶1 Langmuir binding model (A + B = AB). The kinetics data were calculated as K_on_ (association rate), K_off_ (dissociation rate) and K_D_ (K_D_ = K_off_/K_on_). Each SPR run was evaluated based on the recommended range of several statistical measurements provided by the Biacore T200 evaluation software including χ^2^ (measures how closely a model fits the experimental data); U value (indicates if the parameters are uniquely decided); tc (a measure of mass transport limitation); and T-values (analogous to signal-to-noise for the fitted parameter values). All experiments were repeated between 4–9 times.

### Luciferase Assay

Soluble RGMA and HJV (lacking the GPI anchor) fused to the Fc portion of human IgG (RGMA.Fc and HJV.Fc) were generated [4], [19] and purified [19] as previously described. Hepatoma-derived Hep3B cells were transfected with the hepcidin promoter firefly luciferase construct [5] and a control Renilla luciferase vector (pRLTK, Promega). Transfected cells were incubated alone, with BMP ligands (5 ng/ml BMP9, 50 ng/ml BMP5, or 25 ng/ml BMP2, BMP4, BMP6, or BMP7), or with the BMP ligands plus 0.2 to 50 µg/ml RGMA.Fc or HJV.Fc, followed by measurement of relative luciferase activity by dual luciferase assay (Promega) as previously described [19].

### Statistics

Statistical significance was determined by one-way analysis of variance (ANOVA) with the Bonferroni’s or Dunnett’s post-hoc tests for pair-wise multiple comparisons as indicated. Statistical analysis was conducted using Prism 4.0 (La Jolla, CA) statistical software and *P*<0.05 was considered significant.

## Results

### HJV Exhibits Preferential Binding to the BMP6/BMP5/BMP7 Subfamily Compared with RGMA and RGMB, with the Highest Affinity for BMP6

**Figure 1:**
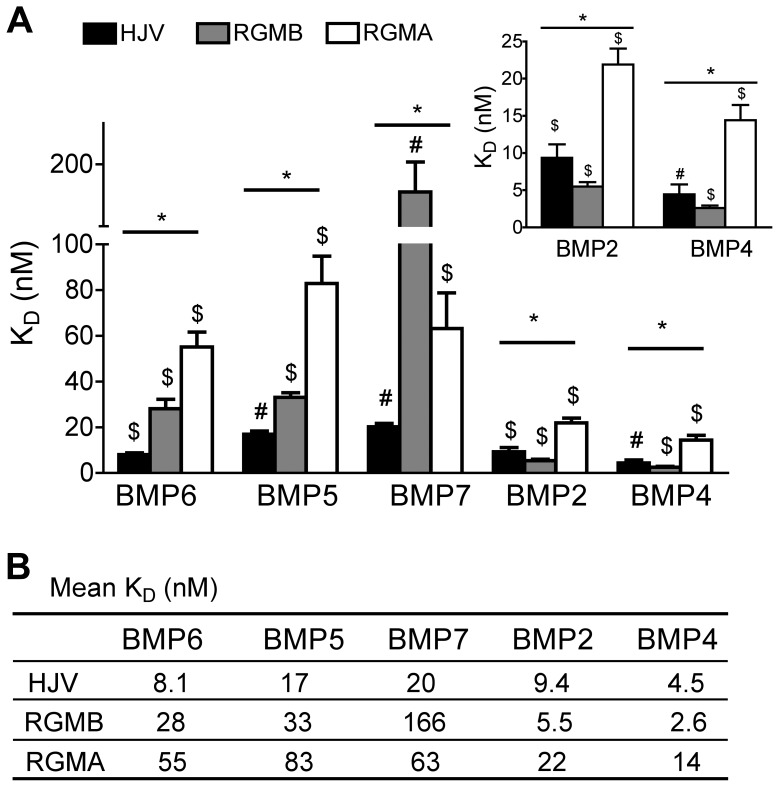
Binding affinity of RGM proteins for BMP ligands. (**A**) The binding affinity (K_D_ = K_off_/K_on_) of each RGM protein for each BMP ligand was measured by surface plasmon resonance and plotted as mean ± SD (n = 4–9 per group). The mean K_D_ ± SD of RGM proteins for BMP2 and BMP4 is also shown as an inset to better demonstrate their lower K_D_ values. The mean K_D_ is reported numerically in (**B**). (**A**) Black bar: HJV; gray bar: RGMB; white bar: RGMA. Statistical significance among 3 RGM proteins for binding to each BMP ligand was determined by one-way analysis of variance (ANOVA) with the Dunnett’s post-hoc test for pair-wise multiple comparisons (* all comparisons are significant (*P*<0.05)). For comparisons among 5 BMP ligands for binding to each RGM protein, one-way ANOVA with the Bonferroni’s post-hoc test was used (# all comparisons are significant (*P*<0.05); $ not all comparisons are significant: for HJV, all pair-wise comparisons between BMP ligands are significant except for BMP2 *vs* BMP6; for RGMB, all pair-wise comparisons between BMP ligands are significant except for BMP2 *vs* BMP4 and BMP5 *vs* BMP6; for RGMA, all pair-wise comparisons between BMP ligands are significant except for BMP2 *vs* BMP4, BMP5 *vs* BMP7 and BMP6 *vs* BMP7.

**Figure 2:**
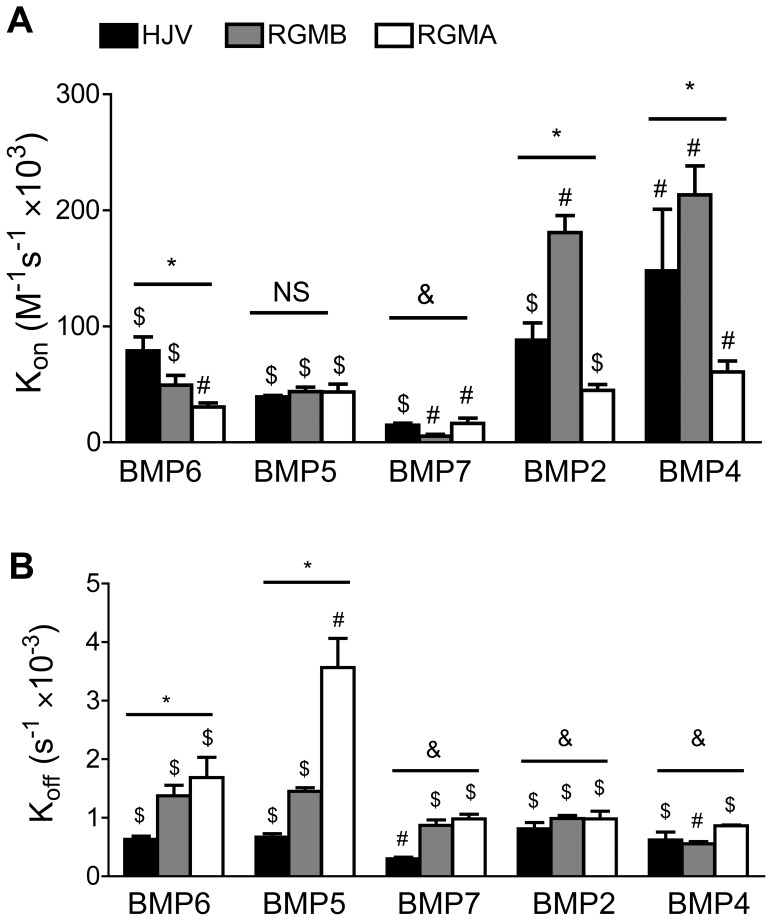
Association rate ( K_on_ **) and dissociation rate (**K_off_ **) between RGM proteins and BMP ligands.** The association rate (K_on_, **A**), and dissociation rate (K_off_, **B**) between each RGM protein and each BMP ligand was measured by SPR and plotted as mean ± SD (n = 4–9 per group). Black bar: HJV; gray bar: RGMB; white bar: RGMA. Statistical significance was determined as described in Figure 1. For comparisons among 3 RGM proteins for each BMP ligand: * all comparisons are significant (*P*<0.05); & not all comparisons are significant, as detailed below; NS all comparisons are not significant (*P*>0.05). For comparisons among 5 BMP ligands for each RGM protein: # all comparisons are significant (*P*<0.05); $ not all comparisons are significant, as detailed below. (**A**) $: For HJV, all pair-wise comparisons between BMP ligands are significant except for BMP2 *vs* BMP6, BMP5 *vs* BMP6, and BMP5 *vs* BMP7; for RGMB, all pair-wise comparisons between BMP ligands are significant except for BMP5 *vs* BMP6; for RGMA, all pair-wise comparisons between BMP ligands are significant except for BMP2 *vs* BMP5. &: For BMP7, all pairwise comparisons are significant except for HJV vs RGMA (**B**) $: For HJV, all pair-wise comparisons between BMP ligands are significant except for BMP2 *vs* BMP5, BMP4 *vs* BMP5, BMP4 *vs* BMP6, and BMP5 *vs* BMP6; for RGMB, all pair-wise comparisons between BMP ligands are significant except for BMP2 *vs* BMP7 and BMP5 *vs* BMP6; for RGMA, all pair-wise comparisons between BMP ligands are significant except for BMP2 *vs* BMP4, BMP2 *vs* BMP7, BMP4 *vs* BMP7, and BMP6 *vs* BMP7. &: For BMP7, all pairwise comparisons are significant except for RGMB *vs* RGMA; for BMP2, all pairwise comparisons are significant except for RGMA *vs* RGMB; for BMP4, all pairwise comparisons are significant except for RGMB *vs* HJV.

We have previously demonstrated that HJV binds to BMP6 and that *Bmp6* knockout mice exhibit an equivalent iron overload phenotype to *Hjv* knockout mice suggesting that BMP6 is an important endogenous ligand for HJV in the regulation of hepcidin expression and systemic iron balance [18], [34]. We therefore quantitated the binding kinetics of HJV to BMP6 using surface plasmon resonance (SPR). Results were compared with the binding of other RGM family members (RGMA and RGMB) to BMP6. Results were also compared to the closely related BMP5 and BMP7 ligands, which together with BMP6, share 71–80% amino acid identity in the mature region and constitute a subfamily of the BMP ligands [43]. Mean K_D_, K_on_, and K_off_ from 4–9 experiments for each interaction are reported in Figure 1 and Figure 2, while representative sensograms are shown in Figure S1, Figure S2, and Figure S3.

As shown in Figure 1, HJV bound to BMP6 with high affinity (K_D_ 8.1 nM). In comparison, RGMB and RGMA bound to BMP6 with about 3.5- and 5-fold lower affinity (K_D_ 28 nM and 55 nM respectively) (Figure 1). The higher affinity of HJV for BMP6 was due to a combination of a faster association rate and slower dissociation rate compared with the other RGM proteins (Figure 2).

HJV bound to BMP5 with about 2-fold lower affinity (K_D_ 17 nM) than BMP6 (Figure 1). This lower affinity was mostly due to a slower association rate of HJV for BMP5 compared with BMP6 (Figure 2). Similar to the results for BMP6, RGMB and RGMA bound to BMP5 with about 2- and 5-fold lower affinity (K_D_ 33 nM and 83 nM respectively) compared with HJV (Figure 1). RGMB binding to BMP5 was similar to BMP6, while the lower affinity of RGMA for BMP5 compared with BMP6 was mostly due to a faster dissociation rate (Figure 2).

All RGM proteins had lower or equivalent affinity for BMP7 compared with BMP6 or BMP5 (Figure 1). This was mainly due to a slower association rate, although RGMs, particularly HJV, also tended to have a slower dissociation rate for BMP7 (Figure 2). These slower kinetics and the need for higher concentrations of analyte yielded poor fitting of BMP7-RGM interactions on CM5 sensor chips, necessitating the use of CM4 sensor chips for these interactions, which yielded reasonable fitting. As a control, we obtained similar results for BMP6-HJV interaction on CM4 sensor chips compared with CM5 sensor chips (data not shown). HJV had the highest affinity for BMP7 among the RGMs (K_D_ 20 nM) followed by RGMA and RGMB (K_D_ 63 nM and 166 nM respectively) (Figure 1). This was mainly due to a very slow dissociation rate of HJV for BMP7 (Figure 2).

### RGMB Exhibits Preferential Binding to the BMP2/BMP4 Subfamily Compared with HJV and RGMA, with the Highest Affinity for BMP4

Next, we tested the binding kinetics of RGM proteins for another main subfamily of BMP ligands, BMP2 and BMP4, which possess 86% amino acid identity to each other, but only 54–60% identity to the BMP6/BMP5/BMP7 subfamily in the mature region [43]. Mean K_D_, K_on_, and K_off_ from 4–9 experiments for each interaction are reported in Figure 1 and Figure 2, while representative sensograms are shown in Figure S4 and Figure S5.

As shown in Figure 1, RGMB had the highest binding affinity for BMP2 among the RGMs (K_D_ 5.5 nM). This was about 5-30-fold higher than the affinity of RGMB for the BMP6/BMP5/BMP7 subfamily (Figure 1), mostly due to a faster association rate (Figure 2). HJV had intermediate binding affinity for BMP2 among the RGM family (K_D_ 9.4 nM), about 1.6-fold lower than RGMB and about 2.5-fold higher than RGMA (Figure 1). In contrast to RGMB, the affinity of HJV for BMP2 was nearly identical to the binding affinity of HJV for BMP6 (Figure 1). Similar to most of the other BMPs tested, RGMA exhibited the lowest binding affinity among the RGMs for BMP2 (K_D_ 22 nM, Figure 1), mostly due to a slower association rate compared with the other RGMs (Figure 2). RGMA was similar to RGMB in that it exhibited preferential binding to BMP2 compared with the BMP6/BMP5/BMP7 subfamily, about 2-4-fold higher (Figure 1). This was due to a combination of a slower dissociation rate and faster association rate of RGMA for BMP2 compared with the BMP6/BMP5/BMP7 subfamily (Figure 2).

The relative affinity of RGM family members for BMP4 was very similar to BMP2 (Figure 1). RGMB had the highest binding affinity for BMP4 (K_D_ 2.6 nM), about 2-fold higher than HJV (K_D_ 4.5 nM) and 5–6-fold higher than RGMA (K_D_ 14 nM) (Figure 1). Interestingly, BMP4 had the highest affinity for all RGM proteins relative to the other BMPs tested (Figure 1), mostly due to a faster association rate, and, to a lesser extent, a slower dissociation rate (Figure 2). The affinity of all of the RGMs for BMP4 was about 2-fold higher than BMP2 (although this difference was not statistically significant for RGMA or RGMB). Similar to the results for BMP2, RGMB exhibited a strong preferential binding to BMP4 compared with the BMP6/BMP5/BMP7 subfamily, about 10- to 64-fold (Figure 1), mostly due to a faster association rate (Figure 2). RGMA exhibited an intermediate preferential binding for BMP4 compared with the BMP6/BMP5/BMP7 subfamily, about 4- to 6-fold (Figure 1), due to a combination of a faster association rate and slower dissociation rate (Figure 2). HJV bound to BMP4 with only about 2-4-fold higher affinity than the BMP6/BMP5/BMP7 subfamily (Figure 1), mostly due to a faster association rate (Figure 2).

### RGM Family Members do not Bind BMP9

BMP9 exhibits 50–55% amino acid identity to BMP2, BMP4, BMP5, BMP6, and BMP7 [44], and has also been demonstrated to stimulate hepcidin expression in liver-derived cells in culture [19]. We therefore tested the binding kinetics of HJV and other RGM family members for BMP9 using SPR. In contrast to the other BMP ligands tested, BMP9 did not bind to any of the RGM family members (Figure S6A–C). As a positive control, BMP9 was able to bind to the soluble portion of the BMP type I receptor ALK1 fused to the Fc region of human IgG (ALK1-Fc, Figure S6D), as previously demonstrated [45].

### Soluble RGM Proteins Fused to the Fc Portion of Human IgG (RGM.Fc) Selectively Inhibit the Biologic Activity of BMP Ligands

To provide independent confirmation of the relative binding affinities of RGM family members for BMP ligands measured by SPR, we tested the ability of soluble RGMA.Fc fusion proteins to inhibit the biologic activity of BMP ligands in a cell culture system. Analogous data have previously been published for HJV.Fc and RGMB.Fc [18]–[19]. The mechanism by which these RGM.Fc fusion proteins inhibit BMP biologic activity is by binding and sequestering BMPs to prevent them from interacting with cell surface BMP receptors [19]. The biologic activity of BMP ligands we studied was the ability to induce hepcidin promoter activity in hepatoma-derived Hep3B cells as measured by dual luciferase assay, which we have previously well characterized [19].

**Figure 3:**
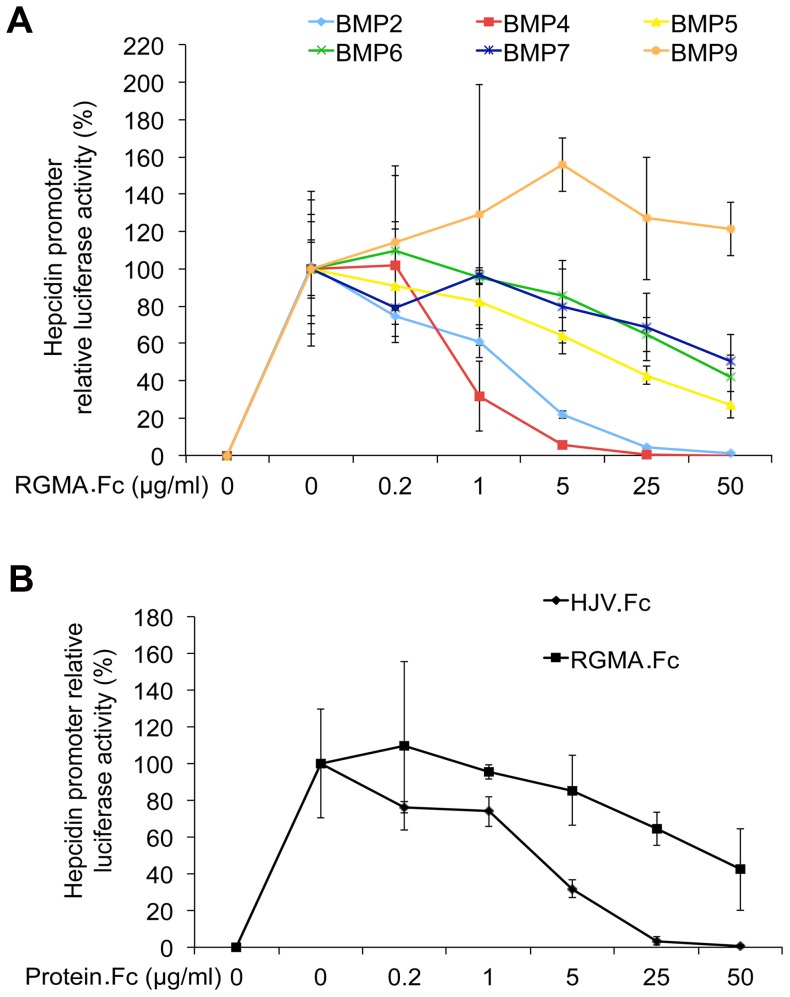
Soluble RGMA.Fc selectively inhibits BMP induction of hepcidin promoter luciferase activity. (**A**) Hep3B cells were transfected with the hepcidin promoter firefly luciferase construct and the control Renilla pRL-TK vector. Transfected cells were incubated alone, with 5 ng/ml BMP9, 50 ng/ml BMP5, or 25 ng/ml BMP2, BMP4, BMP6, or BMP7 ligands, or with the BMP ligands plus 0.2 to 50 µg/ml RGMA.Fc as indicated, followed by measurement of relative luciferase activity. (**B**) Hep3B cells were transfected as indicated in Panel **A**. Transfected cells were incubated alone, with 25 ng/ml BMP6 ligand, or with 25 ng/mL BMP6 ligand plus 0.2 to 50 µg/ml RGMA.Fc or HJV.Fc as indicated followed by measurement of relative luciferase activity. (**A–B**) Results are reported as the mean ± SD of the percent decrease in relative luciferase activity for cells treated with BMP ligands in combination with RGMA.Fc or HJV.Fc compared with cells treated with BMP ligands alone (n = 3–4 per group).

RGMA.Fc inhibited the biologic activity of BMP4 and BMP2 most robustly, with lesser inhibition of BMP5, BMP6, and BMP7, and no inhibition of BMP9 (Figure 3A). In a head-to-head comparison, HJV.Fc was more potent than RGMA.Fc at inhibiting the biologic activity of BMP6 (Figure 3B). These functional data in a cell culture system support the relative binding constants measured by SPR (Figure 1).

## Discussion

Here, we used SPR to perform the first comprehensive quantitative comparison of the binding interactions between the RGM family of BMP co-receptors and BMP ligands. We demonstrated that RGM proteins exhibited a wide range of binding affinities for BMP2, BMP4, BMP5, BMP6, and BMP7 ligands ranging from 2.6 nM to 166 nM, and none of the RGMs bound to BMP9. One characteristic feature shared by all of the RGM proteins is the order of preferential binding to the various BMP ligands. All RGMs exhibited the highest binding affinity for BMP4, followed in order by BMP2, BMP6, BMP5, and BMP7, while none of the RGMs bound to BMP9. However, one notable difference between the RGM proteins is that the binding affinity of HJV for the BMP6/BMP5/BMP7 subfamily was significantly higher than RGMB and RGMA, and was very close to the binding affinity of HJV for BMP4 and BMP2. In contrast, RGMB and to a lesser extent RGMA exhibited a much weaker binding to the BMP6/BMP5/BMP7 subfamily compared with the BMP4/BMP2 subfamily, and compared with HJV.

Binding affinities have previously been reported for the interaction between BMP2 and all RGM family members, and BMP4 binding to RGMA in 4 prior studies [3], [40], [46]–[47]. An important limitation in 3 of these studies is the use of RGM.Fc fusion proteins, which form dimers [3], [40], [46], whereas we used monomeric proteins since HJV is predicted to be monomeric [47]. Therefore, results in these prior studies may be confounded by avidity effects. One prior study measured the binding affinity of monomeric HJV to BMP2 by SPR [47] and reported a 15-fold lower affinity (K_D_ 140 nM) compared to our study. However, Yang *et al.* used an equilibrium based binding assay [47] rather than binding kinetics as in our study, and real equilibrium may not have been achieved in their study.

The relative binding constants measured by SPR in this study are largely consistent with prior studies of the binding of RGM proteins to BMP ligands, and the biologic activity of RGMs and RGM.Fc fusion proteins in cell culture systems [3]–[5], [18]–[19], [40]–[42]. Cell-free iodinated protein interaction and pull-down assays have previously demonstrated that all RGMs bind to BMP2 and BMP4, and that HJV binds to BMP6 [3]–[5], [18]. For all RGMs, excess cold BMP7 was unable to compete for binding to BMP2 and BMP4, suggesting preferential binding of RGMs to BMP2 and BMP4 versus BMP7 [3]–[5], consistent with the results in this study.

Prior studies have also demonstrated that transfection of cDNAs encoding each RGM increased BMP2 and BMP4 signaling, but not BMP7 signaling, in cell culture systems [3]–[5], [40]–[42]. Additionally, transfection of cDNAs encoding HJV, but not RGMA or RGMB, mediated BMP6 signaling in cell culture systems that expressed high levels of endogenous BMP6 [40]–[42]. Thus, all RGMs can mediate BMP2 and BMP4 signaling and HJV can mediate BMP6 signaling in cell culture systems, consistent with the highest binding affinities measured for these interactions by SPR in this study.

Biologic inhibition assays in hepatoma-derived cell cultures using soluble RGM.Fc fusion proteins to inhibit BMP ligand-mediated hepcidin promoter luciferase activity have previously demonstrated that HJV.Fc inhibited BMP4, BMP2, BMP6 and BMP5 most robustly with lesser inhibition of BMP7 and no inhibition of BMP9 [19], while RGMB.Fc inhibited BMP4 and BMP2 most robustly, with lesser inhibition of BMP6, BMP5, and BMP7 and no inhibition of BMP9 [18]. In a head-to-head comparison, HJV.Fc inhibited BMP6 more robustly than RGMB.Fc [18]. Together with the biological inhibition data using RGMA.Fc here (Figure 3), these data overall support the relative binding affinities measured by SPR in this study (Figure 1). These data also provide further insight into a potential therapeutic role for exogenously administered RGM.Fc proteins as selective BMP signaling pathway inhibitors. For example, HJV.Fc has been previously demonstrated to inhibit BMP-mediated hepcidin expression and improve iron availability *in vivo* [18]–[19], [39]. These data may also shed light on a functional role for endogenous forms of soluble RGM proteins as selective BMP pathway inhibitors, particularly HJV, which has been found in measurable levels in circulation [12], [48]–[50].

Interestingly, all RGMs were demonstrated to bind to BMP7 by SPR in the current study, whereas BMP7 was unable to compete with BMP2 and BMP4 for RGM binding in iodinated protein interaction studies [3]–[5], RGMs were not demonstrated to increase BMP7 signaling in cell culture systems [40]–[42], and RGM.Fc proteins demonstrated only a limited ability to inhibit the biological activity of BMP7 in hepcidin promoter luciferase cell culture assays (Figure 3) [18]–[19]. This was particularly notable for HJV, where the overall affinity of the HJV-BMP7 interaction was only about 2.5-fold lower than that for the HJV-BMP6 interaction by SPR, while HJV.Fc was much less potent at inhibiting BMP7 compared with BMP6 activity in the hepcidin promoter luciferase assays [19]. One explanation for these differences is the slow kinetics of the RGM-BMP7 interactions. The reason for the relatively higher overall affinity for the HJV-BMP7 interaction measured by SPR was the very slow dissociation rate. However, all RGM-BMP7 interactions had a very slow association rate, and this slow association rate may have been the limiting factor precluding any apparent biologic activity of RGM-BMP7 interactions in the binding competition and cell culture assays. Second, there may have been avidity effects from the use of dimeric RGM.Fc fusion proteins in the biologic inhibition assays, while RGM monomers were used in SPR. Additionally, the binding characteristics of RGMs to BMP ligands in solution in the biologic assays may be different compared with BMP ligands that are covalently bound to sensor chips in SPR. Finally, the inability of BMP7 to compete for BMP2 and BMP4 binding could also be explained by nonoverlapping binding sites for BMP7 and BMP2/BMP4 on RGMs. Future studies will be needed to delineate the precise BMP binding domain(s) on RGMs, and whether the ability of RGMs to bind BMP7 measured by SPR correlates with any biologic activity.

Among the RGMs, HJV had the highest binding affinity for BMP6 at 8.1 nM. This is consistent with data from a biologic inhibition assay showing that soluble HJV.Fc fusion protein inhibited BMP6 induction of hepcidin promoter luciferase activity more strongly than RGMB.Fc [19] and RGMA.Fc (Figure 3). This is also consistent with the known physiologic role of HJV and BMP6 *in vivo* in regulating liver hepcidin expression and systemic iron balance [5], [7], [18]–[21], [34]. Indeed, it is not surprising that HJV expression is critical for BMP6-mediated induction of hepcidin in response to iron given the relatively low affinity (K_D_ 1.6 to 39 µM) of BMP6 for the BMP type I and type II receptors that have been demonstrated to be involved in this process [41], [51]. We hypothesize that expression of HJV in hepatocytes enables these cells to respond specifically to low levels of BMP6 that are produced in the liver in response to iron to stimulate hepcidin expression. The significantly lower affinity of RGMB and RGMA for BMP6 may help explain why these proteins cannot compensate for the loss of HJV in regulating hepcidin expression and systemic iron balance. Of note, HJV bound to BMP2 and BMP4 with equal or 2-fold higher affinity than BMP6. Additionally, HJV also exhibited preferential binding to BMP5 and BMP7 compared with RGMB and RGMA. These data raise the possibility that HJV could also have a role in regulating signaling by binding to one or more of these other BMP ligands. Interestingly, BMP2 was recently postulated to have a role in upregulating hepcidin expression in multiple myeloma [52].

RGMB knockout mice exhibit early postnatal death, confirming an important biologic function for RGMB, but the cause of this premature death is still poorly understood [32]. RGMB has also been implicated in axonal regeneration after injury [33], the regulation of inflammatory cytokine expression in immune cells [32], and renal tubule tight junction formation and transepithelial resistance [42], all of which appear to be mediated by RGMB’s BMP signaling function [32]–[33], [42]. Notably, BMP4 and BMP2 ligands have been implicated in these processes [32]–[33], [42], consistent with the preferential affinity of RGMB for BMP4 and BMP2 compared with HJV and RGMA and the other BMP ligands.

RGMA has been demonstrated to mediate repulsive axonal guidance (giving rise to the family name), neural tube closure, neuronal differentiation, cell survival, inflammation and immune cell function [2], [6], [9], [22]–[31]. Many of these actions depend on an interaction with neogenin, a homologue of the netrin receptor deleted in colon cancer [23]–[24], [26]–[30], while the BMP signaling function of RGMA has not been clearly linked to its observed biologic actions. Notably, RGMA exhibited the lowest affinity for most of the BMP ligands tested relative to other RGM family members, with the exception of slightly higher affinity for BMP7 compared with RGMB (which were the 2 lowest affinity interaction measured). Thus, RGMA may have a less prominent role as a BMP co-receptor compared with the other RGM proteins. Nevertheless, RGMA still exhibits a binding affinity as low as 14 nM to 23 nM to the BMP4/BMP2 subfamily, which leaves open a possible physiologic role for the BMP signaling function of RGMA.

In summary, the members of the RGM family of BMP co-receptors exhibit preferential binding for different subsets of BMP ligands. Relative to other RGMs, HJV has the highest affinity for binding to BMP6, consistent with the important physiologic role of HJV-mediated BMP6 signaling in regulating iron homeostasis. These data provide important insights into mechanisms by which BMP signals are finely tuned to exert cell-specific effects.

## Supporting Information

Figure S1 **Representative sensograms of kinetics experiments between BMP6 and RGM proteins by SPR. (A)** HJV protein was diluted in running buffer HBS-EP+ into a series of concentration (6.25, 12.5, 25, 50 and 100 nM) and injected through CM5 chip immobilized with BMP6 at a density of 87.5 RU. **(B)** RGMB protein was diluted in running buffer HBS-EP+ into a series of concentration (18.75, 37.5, 75, 150 and 300 nM) and injected through CM5 chip immobilized with BMP6 at a density of 287.3 RU. **(C)** RGMA protein was diluted in running buffer HBS-EP+ into a series of concentration (30, 75, 150, 300 and 600 nM) and injected through CM5 chip immobilized with BMP6 at a density of 150 RU. **(A–C)** Color lines represent the fitted curves plotted from the 1∶1 Langmuir binding model and the black line represents the experimental curves. The kinetics data (K_D_, K_on_, and K_off_) and quality control parameters (χ^2^, U, T(K_on_) and T(K_off_)) are shown in the tables below each sensogram.
(TIF)

Figure S2 **Representative sensograms of kinetics experiments between BMP5 and RGM proteins by SPR. (A)** HJV protein was diluted in running buffer HBS-EP+ into a series of concentration (10, 20, 40, 80 and 160 nM) and injected through CM5 chip immobilized with BMP5 at a density of 287.4 RU. **(B)** RGMB protein was diluted in running buffer HBS-EP+ into a series of concentration (12.5, 25, 50, 100 and 200 nM) and injected through CM5 chip immobilized with BMP5 at a density of 527.5 RU. **(C)** RGMA protein was diluted in running buffer HBS-EP+ into a series of concentration (50, 100, 200, 400 and 800 nM) and injected through CM5 chip immobilized with BMP5 at a density of 436.1 RU. **(A–C)** Color lines represent the fitted curves plotted from the 1∶1 Langmuir binding model and the black line represents the experimental curves. The kinetics data (K_D_, K_on_, and K_off_) and quality control parameters (χ^2^, U, T(K_on_) and T(K_off_)) are shown in the tables below each sensogram.
(TIF)

Figure S3 **Representative sensograms of kinetics experiments between BMP7 and RGM proteins by SPR. (A)** HJV protein was diluted in running buffer HBS-EP+ into a series of concentration (50, 100, 200, 400 and 800 nM) and injected through CM4 chip immobilized with BMP7 at a density of 284.9 RU. **(B)** RGMB protein was diluted in running buffer HBS-EP+ into a series of concentration (18.75, 37.5, 75, 150 and 300 nM) and injected through CM4 chip immobilized with BMP7 at a density of 365.6 RU. **(C)** RGMA protein was diluted in running buffer HBS-EP+ into a series of concentration (75, 150, 300, 600 and 1200 nM) and injected through CM4 chip immobilized with BMP7 at a density of 286.9 RU. **(A–C)** Color lines represent the fitted curves plotted from the 1∶1 Langmuir binding model and the black line represents the experimental curves. The kinetics data (K_D_, K_on_, and K_off_) and quality control parameters (χ^2^, U, T(K_on_) and T(K_off_)) are shown in the tables below each sensogram.
(TIF)

Figure S4 **Representative sensograms of kinetics experiments between BMP2 and RGM proteins by SPR. (A)** HJV protein was diluted in running buffer HBS-EP+ into a series of concentration (6.25, 12.5, 25, 50 and 100 nM) and injected through CM5 chip immobilized with BMP2 at a density of 118 RU. **(B)** RGMB protein was diluted in running buffer HBS-EP+ into a series of concentration (2, 4, 8, 15 and 30 nM) and injected through CM5 chip immobilized with BMP2 at a density of 198.5 RU. **(C)** RGMA protein was diluted in running buffer HBS-EP+ into a series of concentration (10, 20, 40, 80 and 160 nM) and injected through CM5 chip immobilized with BMP2 at a density of 198.5 RU. **(A–C)** Color lines represent the fitted curves plotted from the 1∶1 Langmuir binding model and the black line represents the experimental curves. The kinetics data (K_D_, K_on_, and K_off_) and quality control parameters (χ^2^, U, T(K_on_) and T(K_off_)) are shown in the tables below each sensogram.
(TIF)

Figure S5 **Representative sensograms of kinetics experiments between BMP4 and RGM proteins by SPR. (A)** HJV protein was diluted in running buffer HBS-EP+ into a series of concentration (5, 10, 20, 40 and 80 nM) and injected through CM5 chip immobilized with BMP4 at a density of 40.2 RU. **(B)** RGMB protein was diluted in running buffer HBS-EP+ into a series of concentration (2, 4, 8, 16 and 32 nM) and injected through CM5 chip immobilized with BMP4 at a density of 96.1 RU. **(C)** RGMA protein was diluted in running buffer HBS-EP+ into a series of concentration (10, 20, 40, 80 and 160 nM) and injected through CM5 chip immobilized with BMP4 at a density of 95.2 RU. **(A–C)** Color lines represent the fitted curves plotted from the 1∶1 Langmuir binding model and the black line represents the experimental curves. The kinetics data (K_D_, K_on_, and K_off_) and quality control parameters (χ^2^, U, T(K_on_) and T(K_off_)) are shown in the tables below each sensogram.
(TIF)

Figure S6 **Representative sensograms of kinetics experiments between BMP9 and RGM proteins, and BMP9 binding to ALK1-Fc by SPR. (A)** HJV protein was diluted in running buffer HBS-EP+ into a series of concentration (10, 25, 50, 100 and 200 nM) and injected through CM5 chip immobilized with BMP9 at a density of 139.1 RU. **(B)** RGMB protein was diluted in running buffer HBS-EP+ into a series of concentration (2, 4, 8, 15 and 30 nM) and injected through CM5 chip immobilized with BMP9 at a density of 160.7 RU. **(C)** RGMA protein was diluted in running buffer HBS-EP+ into a series of concentration (6, 12, 25, 50 and 100 nM) and injected through CM5 chip immobilized with BMP9 at a density of 160.7 RU. **(A–C)** No significant binding was detected and kinetics data cannot be calculated. **(D)** ALK1-Fc protein was diluted in running buffer HBS-EP+ into 100 nM and injected through CM5 chip immobilized with BMP9 at a density of 160.7 RU.
(TIF)
