# Supplementary figures and images for: Repulsive Guidance Molecule (RGM) Family Proteins Exhibit Differential Binding Kinetics for Bone Morphogenetic Proteins (BMPs)
Source: PLoS One. 2012 Sep 27;7(9):e46307. doi: 10.1371/journal.pone.0046307 (PMC3459908; doi:10.1371/journal.pone.0046307)

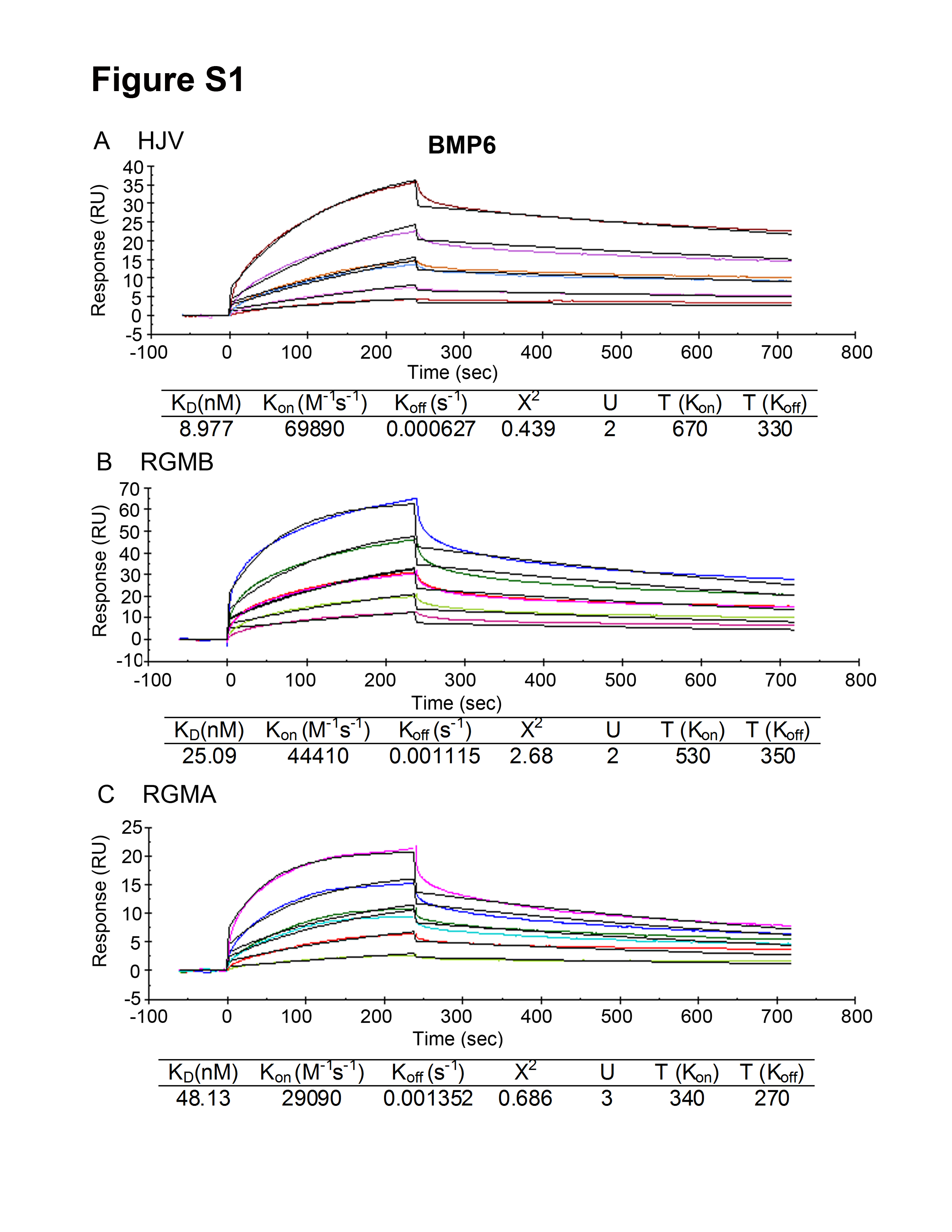

Supplement: Figure S1 — Representative sensograms of kinetics experiments between BMP6 and RGM proteins by SPR. (A) HJV protein was diluted in running buffer HBS-EP+ into a series of concentration (6.25, 12.5, 25, 50 and 100 nM) and injected through CM5 chip immobilized with BMP6 at a density of 87.5 RU. (B) RGMB protein was diluted in running buffer HBS-EP+ into a series of concentration (18.75, 37.5, 75, 150 and 300 nM) and injected through CM5 chip immobilized with BMP6 at a density of 287.3 RU. (C) RGMA protein was diluted in running buffer HBS-EP+ into a series of concentration (30, 75, 150, 300 and 600 nM) and injected through CM5 chip immobilized with BMP6 at a density of 150 RU. (A–C) Color lines represent the fitted curves plotted from the 1∶1 Langmuir binding model and the black line represents the experimental curves. The kinetics data (KD, Kon, and Koff) and quality control parameters (χ2, U, T(Kon) and T(Koff)) are shown in the tables below each sensogram. (TIF) [file pone.0046307.s001.tif]

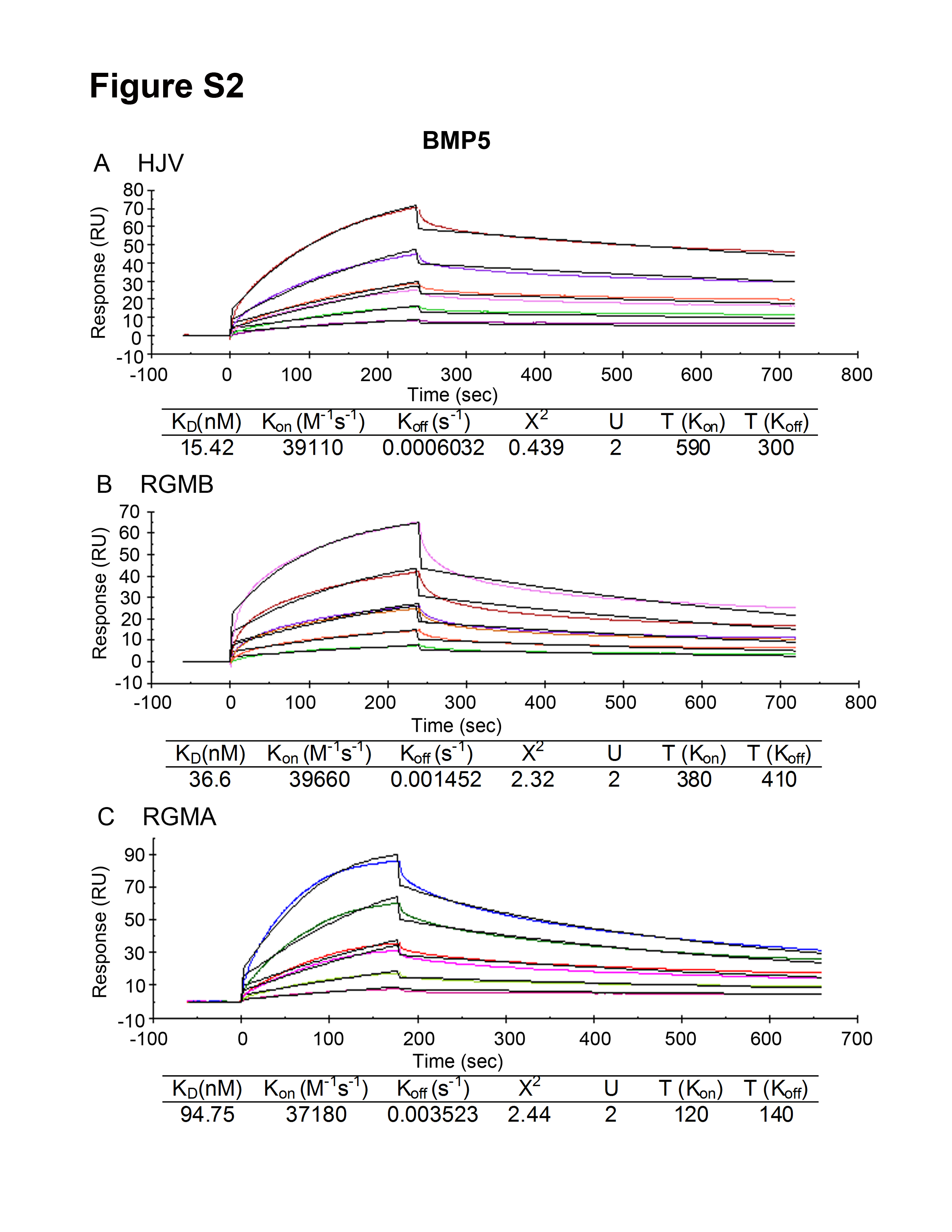

Supplement: Figure S2 — Representative sensograms of kinetics experiments between BMP5 and RGM proteins by SPR. (A) HJV protein was diluted in running buffer HBS-EP+ into a series of concentration (10, 20, 40, 80 and 160 nM) and injected through CM5 chip immobilized with BMP5 at a density of 287.4 RU. (B) RGMB protein was diluted in running buffer HBS-EP+ into a series of concentration (12.5, 25, 50, 100 and 200 nM) and injected through CM5 chip immobilized with BMP5 at a density of 527.5 RU. (C) RGMA protein was diluted in running buffer HBS-EP+ into a series of concentration (50, 100, 200, 400 and 800 nM) and injected through CM5 chip immobilized with BMP5 at a density of 436.1 RU. (A–C) Color lines represent the fitted curves plotted from the 1∶1 Langmuir binding model and the black line represents the experimental curves. The kinetics data (KD, Kon, and Koff) and quality control parameters (χ2, U, T(Kon) and T(Koff)) are shown in the tables below each sensogram. (TIF) [file pone.0046307.s002.tif]

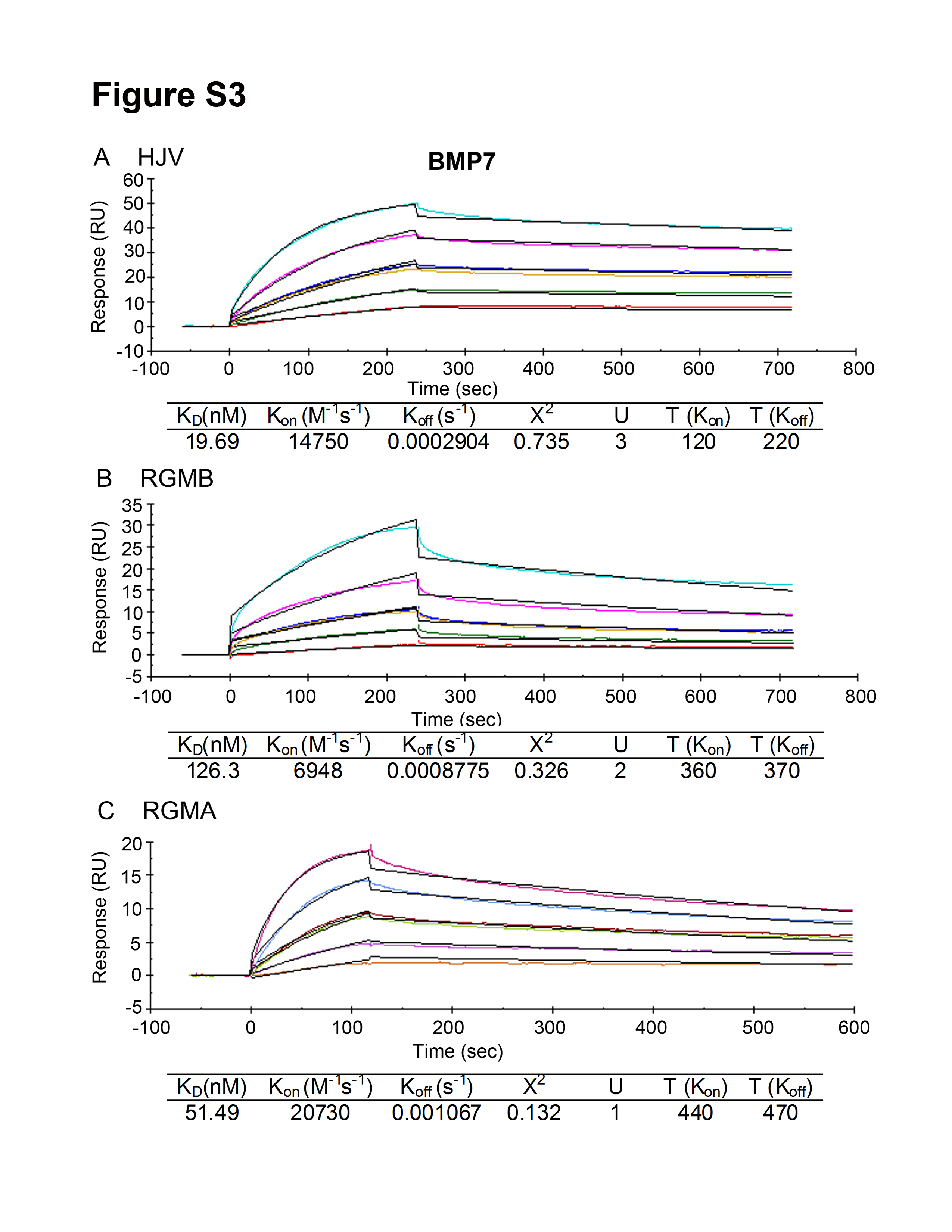

Supplement: Figure S3 — Representative sensograms of kinetics experiments between BMP7 and RGM proteins by SPR. (A) HJV protein was diluted in running buffer HBS-EP+ into a series of concentration (50, 100, 200, 400 and 800 nM) and injected through CM4 chip immobilized with BMP7 at a density of 284.9 RU. (B) RGMB protein was diluted in running buffer HBS-EP+ into a series of concentration (18.75, 37.5, 75, 150 and 300 nM) and injected through CM4 chip immobilized with BMP7 at a density of 365.6 RU. (C) RGMA protein was diluted in running buffer HBS-EP+ into a series of concentration (75, 150, 300, 600 and 1200 nM) and injected through CM4 chip immobilized with BMP7 at a density of 286.9 RU. (A–C) Color lines represent the fitted curves plotted from the 1∶1 Langmuir binding model and the black line represents the experimental curves. The kinetics data (KD, Kon, and Koff) and quality control parameters (χ2, U, T(Kon) and T(Koff)) are shown in the tables below each sensogram. (TIF) [file pone.0046307.s003.tif]

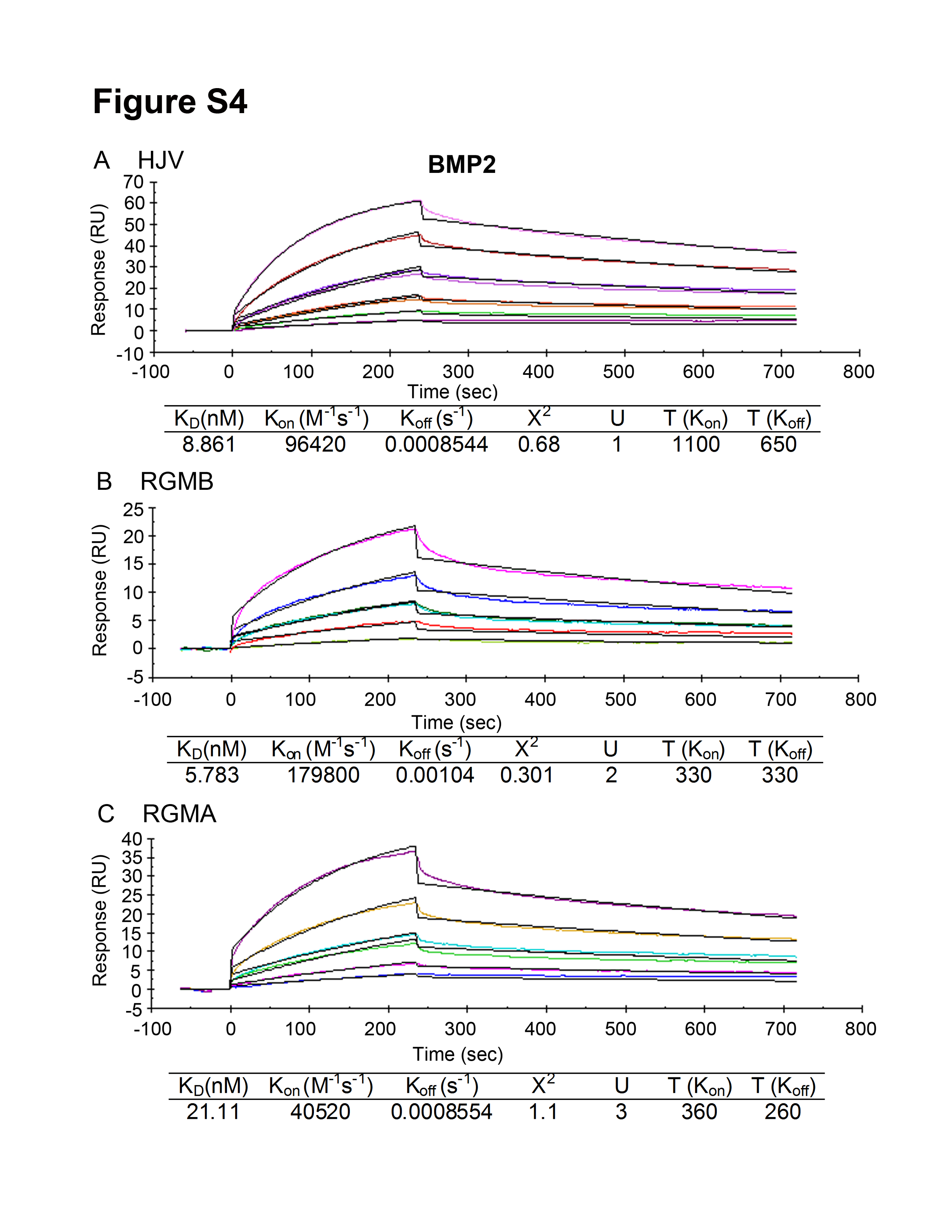

Supplement: Figure S4 — Representative sensograms of kinetics experiments between BMP2 and RGM proteins by SPR. (A) HJV protein was diluted in running buffer HBS-EP+ into a series of concentration (6.25, 12.5, 25, 50 and 100 nM) and injected through CM5 chip immobilized with BMP2 at a density of 118 RU. (B) RGMB protein was diluted in running buffer HBS-EP+ into a series of concentration (2, 4, 8, 15 and 30 nM) and injected through CM5 chip immobilized with BMP2 at a density of 198.5 RU. (C) RGMA protein was diluted in running buffer HBS-EP+ into a series of concentration (10, 20, 40, 80 and 160 nM) and injected through CM5 chip immobilized with BMP2 at a density of 198.5 RU. (A–C) Color lines represent the fitted curves plotted from the 1∶1 Langmuir binding model and the black line represents the experimental curves. The kinetics data (KD, Kon, and Koff) and quality control parameters (χ2, U, T(Kon) and T(Koff)) are shown in the tables below each sensogram. (TIF) [file pone.0046307.s004.tif]

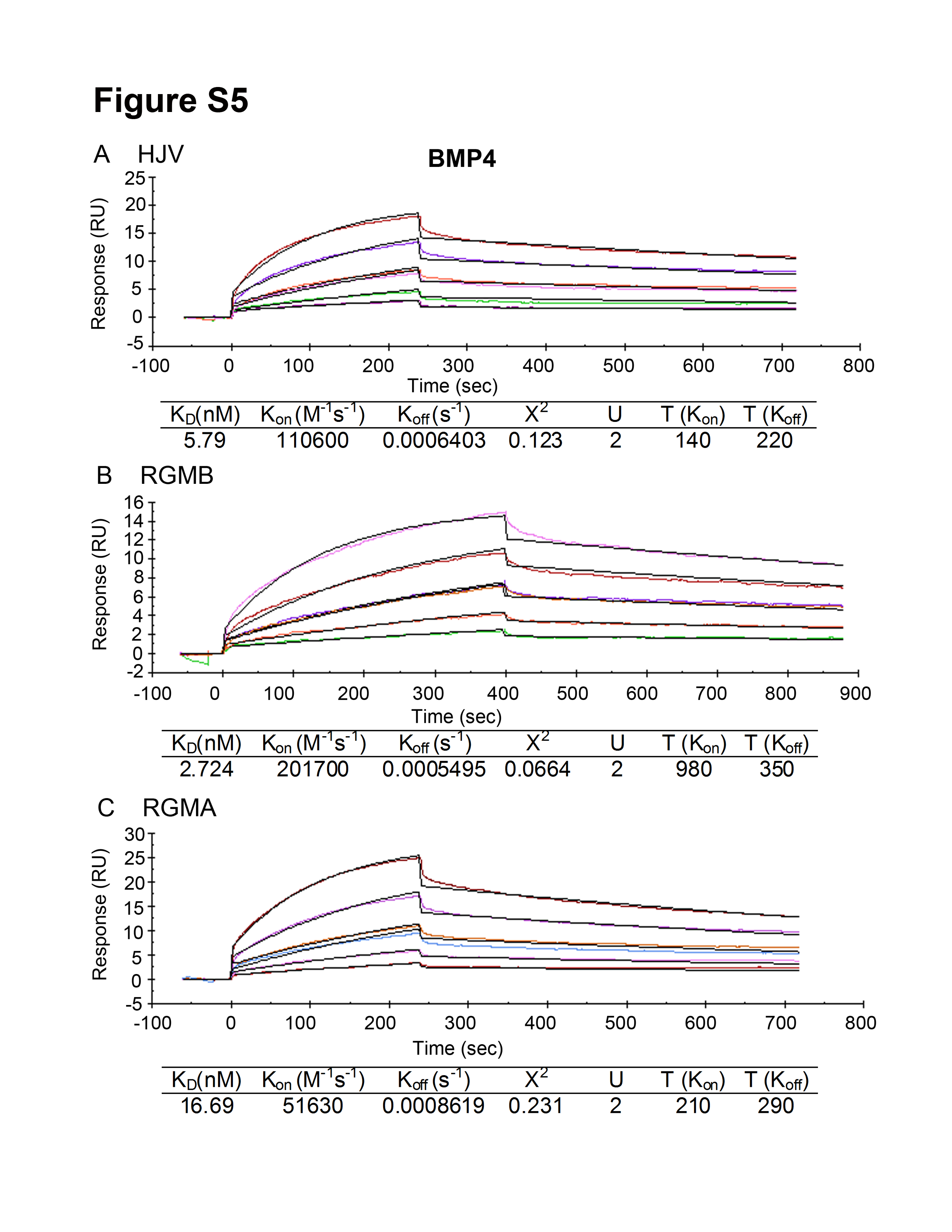

Supplement: Figure S5 — Representative sensograms of kinetics experiments between BMP4 and RGM proteins by SPR. (A) HJV protein was diluted in running buffer HBS-EP+ into a series of concentration (5, 10, 20, 40 and 80 nM) and injected through CM5 chip immobilized with BMP4 at a density of 40.2 RU. (B) RGMB protein was diluted in running buffer HBS-EP+ into a series of concentration (2, 4, 8, 16 and 32 nM) and injected through CM5 chip immobilized with BMP4 at a density of 96.1 RU. (C) RGMA protein was diluted in running buffer HBS-EP+ into a series of concentration (10, 20, 40, 80 and 160 nM) and injected through CM5 chip immobilized with BMP4 at a density of 95.2 RU. (A–C) Color lines represent the fitted curves plotted from the 1∶1 Langmuir binding model and the black line represents the experimental curves. The kinetics data (KD, Kon, and Koff) and quality control parameters (χ2, U, T(Kon) and T(Koff)) are shown in the tables below each sensogram. (TIF) [file pone.0046307.s005.tif]

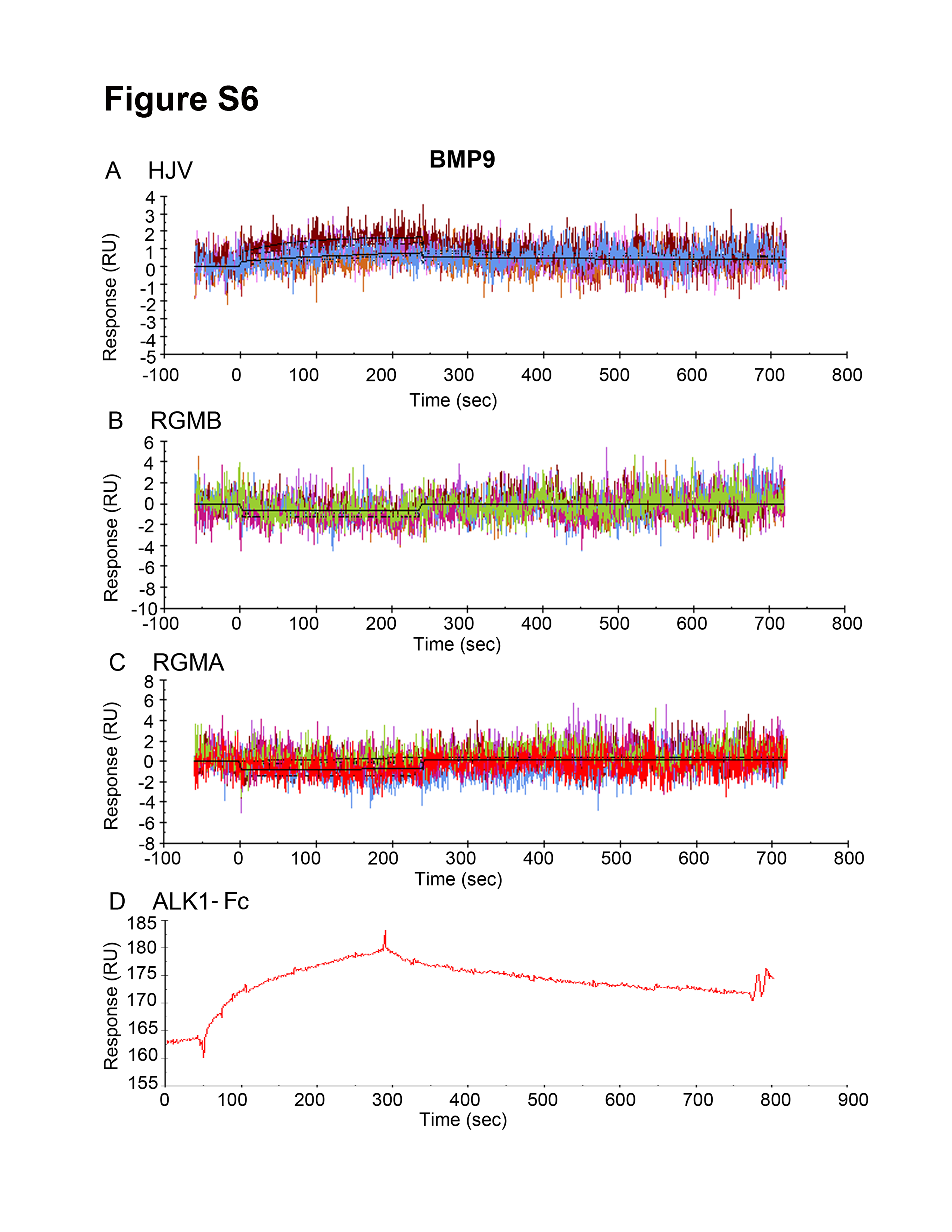

Supplement: Figure S6 — Representative sensograms of kinetics experiments between BMP9 and RGM proteins, and BMP9 binding to ALK1-Fc by SPR. (A) HJV protein was diluted in running buffer HBS-EP+ into a series of concentration (10, 25, 50, 100 and 200 nM) and injected through CM5 chip immobilized with BMP9 at a density of 139.1 RU. (B) RGMB protein was diluted in running buffer HBS-EP+ into a series of concentration (2, 4, 8, 15 and 30 nM) and injected through CM5 chip immobilized with BMP9 at a density of 160.7 RU. (C) RGMA protein was diluted in running buffer HBS-EP+ into a series of concentration (6, 12, 25, 50 and 100 nM) and injected through CM5 chip immobilized with BMP9 at a density of 160.7 RU. (A–C) No significant binding was detected and kinetics data cannot be calculated. (D) ALK1-Fc protein was diluted in running buffer HBS-EP+ into 100 nM and injected through CM5 chip immobilized with BMP9 at a density of 160.7 RU. (TIF) [file pone.0046307.s006.tif]
